# Virus-specific T-cells from third party or transplant donors for treatment of EBV lymphoproliferative diseases arising post hematopoietic cell or solid organ transplantation

**DOI:** 10.3389/fimmu.2023.1290059

**Published:** 2024-01-11

**Authors:** Richard J. O’Reilly, Susan Prockop, Joseph H. Oved

**Affiliations:** ^1^ Department of Pediatrics, Stem Cell Transplantation and Cellular Therapies Service, Memorial Sloan Kettering Cancer Center, New York, NY, United States; ^2^ Pediatric Stem Cell Transplantation, Boston Children’s Hospital/Dana-Farber Cancer Institute, Boston, MA, United States

**Keywords:** third party, Epstein-Barr virus (EBV), T-cells, lymphoma, transplantation, EBV-CTLs, lymphoproliferative disorder

## Abstract

EBV^+^ lymphomas constitute a significant cause of morbidity and mortality in recipients of allogeneic hematopoietic cell (HCT) and solid organ transplants (SOT). Phase I and II trials have shown that in HCT recipients, adoptive transfer of EBV-specific T-cells from the HCT donor can safely induce durable remissions of EBV^+^ lymphomas including 70->90% of patients who have failed to respond to treatment with Rituximab. More recently, EBV-specific T-cells generated from allogeneic 3^rd^ party donors have also been shown to induce durable remission of EBV^+^ lymphomas in Rituximab refractory HCT and SOT recipients. In this review, we compare results of phase I and II trials of 3^rd^ party and donor derived EBV-specific T-cells. We focus on the attributes and limitations of each product in terms of access, safety, responses achieved and durability. The limited data available regarding donor and host factors contributing to T cell persistence is also described. We examine factors contributing to treatment failures and approaches to prevent or salvage relapse. Lastly, we summarize strategies to further improve results for virus-specific immunotherapies for post-transplant EBV lymphomas.

## Introduction

Oligoclonal and monoclonal EBV-induced lymphoproliferative diseases are the cause of significant morbidity and mortality in recipients of allogenic solid organ (SOT) and hematopoietic cell transplants (HCT) (1–5).

In SOT recipients, post-transplant lymphoproliferative disorders (PTLD) presenting with polyclonal lymphoid hyperplasia usually respond to reductions of immunosuppressive drug doses (5). However, patients presenting with monoclonal/monomorphic lymphomas rarely respond to reduced immunosuppression. In a recent survey of 5, 365 SOTs in the United Kingdom by Santarsieri et al. (6), the cumulative risk of these EBV lymphomas at 5 years was 1-3% for single SOTs but 18% for multiorgan grafts and up to 10% for kidney grafts at 20 years. Of these lymphomas, 65% were EBV+. While treatment with Rituximab +/- multi-agent chemotherapy such as CHOP can induce complete remissions in 40-50% of SOT recipients, relapses are common (7–9). Furthermore, treatment related mortality associated with R-CHOP ranges from 6-30%.

In HCT recipients, PTLD usually occur within 1 year post transplant and are almost always EBV+. In our cohort of patients treated with HCT or 3^rd^ party donor EBVCTLs, 77/79 (97%) presented as monomorphic lymphomas; 87% as DLBCL (10, 11). In analyses of large patient cohorts, several factors have been consistently identified to contribute to risk of developing PTLD following HCTs specifically: HCTs from HLA disparate donors, HCTs from EBV seropositive donors to seronegative hosts, and HCTs depleted of T-cells by *in vitro* selection or *in vivo* with anti-thymocyte globulin (12–15). Their incidence, by histocompatibility, varies from 1.2% to 3.2% for HCT from related, HLA-matched *vs*. mismatched donors, respectively; and 4% to 11% for HCT from unrelated donors that are matched or mismatched (12). These lymphomas progress rapidly. Rituximab administered as pre-emptive treatment to patients with a persistently high or escalating levels of EBV DNA in the blood can induce clearance of EBV DNA and prevent EBVLPD in 89.7% or more patients (12, 16–18) and can also induce remissions of early PTLD in 75% of patients (12). However, for high-risk patients (e.g. patients > 30 years old, with extranodal disease or ongoing GVHD on immununosuppressive drugs) this treatment is often inadequate, with a cumulative risk of PTLD-related mortality of over 70% within 2 months post diagnosis (12). Preemptive Rituxan treatment may also be complicated by transient neutropenia and prolonged impairment of B-cell reconstitution (19). In recipients of HLA haploidentical T-cell depleted grafts treated for EBVLPD with Rituxan, relapses of EBVLPD lacking expression of CD20 have also been observed (20). While combination chemotherapy can be active, its morbidity and mortality in allogeneic HCT patients is excessive (21).

In 1994, our group first reported 5 patients with monoclonal EBVPTLD who achieved durable complete remissions (CR) after adoptive transfer of unselected lymphocytes from their seropositive HCT donors (22). Subsequently, Rooney et al. (23) reported the potential of *in vitro* generated allo-HCT donor-derived EBV-specific T cells to induce remissions of PTLD without GVHD. Since then, several studies have confirmed these findings (10, 24–27). Our group has reported CRs in 13 of 19 patients (68%) with documented EBV+ lymphomas following treatment with alloHCT donor derived EBVCTL, compared to 18/30 (73%) who achieved CR or durable PR following DLI (10). Of those treated with DLI, 14% developed grade 2-3 acute or chronic GVHD. In contrast, no recipient of donor derived EBVCTL developed GVHD, reflecting the depletion of alloresponsive T cells that occurs over 28-35 days of *in vitro* culture after sensitization with autologous EBVCTLs (10).

To date, application of transplant donor derived T cells in adoptive therapy has been limited. This is due, to the logistical challenges of isolating and/or generating EBV-specific T cells fast enough to treat the rapidly progressive EBV+ lymphomas that develop. Generation of EBVCTLs may also not be possible for alloHCT recipients if the HCT donor is seronegative or a cord blood graft. Similarly, autologous EBVCTLs may not be expandable from previously seronegative organ allograft recipients on immunosuppressive drugs.

To address these limitations, we and several other groups have been exploring an approach initially introduced by Haque et al. (28, 29), namely, the use of EBV-specific CTLs generated from healthy seropositive 3^rd^ party donors, (i.e. donors other than the donor of the patient’s transplant). In this review, we will comparatively examine the results of reported Phase I and II trials of transplant donor and 3^rd^ party donor EBV-specific T-cells in the treatment of EBV lymphomas arising after an allogenic HCT or SOT. We will evaluate attributes of the patient’s lymphoma and of the 3^rd^ party or transplant donor-derived EBV-specific T-cells that contribute to treatment responses and their durability as well as those associated with treatment failure. Lastly, we will examine currently available and developing approaches to salvage treatment failures.

## HCT donor and 3^rd^ party donor-derived EBV-specific T-cells: their generation and properties

EBV seropositive HCT donor-derived and 3^rd^ party-derived EBVCTLs currently in clinical trials can be generated from heparinized blood samples or from standard or G-CSF mobilized leukaphereses (10, 17–23, 26, 30). The EBVCTLs produced are predominantly polyclonal T-cells generated *in vitro* in response to autologous EBVBLCLs (10, 23–29), autologous EBVBLCLs engineered to also express other viral proteins (31, 32) or mononuclear cells loaded with pools of immunogenic peptides from latency 1-3 EBV proteins alone or together with peptides from CMV, adenovirus, BK virus +/- others for multivirus-specific T-cells (33–35). Alternatively, the EBVCTLs have been isolated after brief exposure to these peptide pools by immunoadsorption based on their production of IFNγ or expression of a T-cell activation marker indicating a response (36–39).

The T-cells generated by these techniques vary depending on their donor, the conditions and incubation times employed for their *in vitro* expansion or for their early isolation and particularly, the antigenic stimulus used for sensitization. The cited reports can be consulted to ascertain these specifics. T-cells selectively sensitized with autologous EBVBLCLs or EBV peptide pools loaded on autologous monocyte-derived dendritic cells are usually predominantly CD8+ T-cells (10, 11, 23, 28, 29). T-cells sensitized with autologous APCs loaded with immunogenic peptides from multiple viruses usually have higher proportions of CD4+ T-cells. This phenomenon likely reflects the higher proportion of CD4+ T-cells responsive to dominant adenoviral hexon5 peptides presented by class II HLA alleles (31). The multivirus peptide sensitized IFNγ^+^ T-cells isolated by immunomagnetic bead adsorption, usually contain near equal proportions of CD4+ and CD8+ T-cells. The EBV-specific CD8+ T-cells are predominantly T_EM_ but, interestingly, always contain a significant proportion of Tcm even after extended culture (10, 11); the CD4+ T-cells are TH1 type. In all cases both the CD8+ and CD4+ EBV-specific T-cells are cytolytic.

Banked 3^rd^ party donor-derived T-cells cleared for adoptive therapy are routinely assessed for viral specificity and to ascertain their lack of *in vitro* alloreactivity against fully allogeneic targets. The frequency of T-cells specific for the sensitizing peptides is usually measured by ELISpot assay, limiting dilution analysis of CTL precursors (CTLp), or quantitation of T-cells generating intracellular cytokine in response to specific peptides (10, 11, 36, 37, 39). If the epitope specificities and HLA restrictions of the adoptively transferred EBV-specific T-cells are already identified EBV epitope specific T-cells expanding in the patient can also be measured by FACS-based quantitation of unstimulated T-cells binding the peptide epitope/HLA multimers (30, 35). Furthermore, the use of such multimers can distinguish HLA partially matched 3^rd^ party T-cells-specific for EBV epitopes presented by shared HLA alleles from endogenous T-cells restricted by alleles unique to the patient, the HCT donor or 3^rd^ party donor and can provide estimates of their relative contributions to initial and long-term reconstitution of EBV-specific immunity (36, 37, 40).

The epitope specificities and HLA restrictions of 3^rd^ party donor-derived EBVCTLs, like those from HCT donors vary significantly but usually reflect the dominant EBV-specific T-cells maintained in a latently infected, seropositive healthy donor after resolution of a primary infection. The population of EBV-specific T-cells maintained in the blood is large (5.7-33 CTL precursors/10^6^ PBMNC) (10). It is usually specific for a limited repertoire of latent and lytic phase EBV epitopes presented by prevalent HLA alleles in the donor’s HLA genotype (41). However, responses to each of these immunodominant epitopes is polyclonal, each T-cell clone expressing a distinct T-cell receptor specific for the epitope bound to its presenting HLA allele. After sensitization with a pool of defined EBV peptides, these epitope-specific T-cells can be isolated by their binding to epitope-HLA multimers, and their T-cell receptors sequenced (40, 42). The number of high frequency T-cell clonotypes expressing distinctive TCRs responding to a specific immunodominant epitope varies, but in one study ranged from 9-40 (40). Each of the epitope-specific T-cell clonotypes in this repertoire can be characterized as to phenotype and effector function. Following adoptive transfer, the expansion of each epitope-specific T-cell can be sequentially quantitated. Differential expansions of specific clonotypes have also been correlated with clinical response (40, 42, 43). Ultimately, this may identify epitope-specific TCRs characterizing T-cell clonotypes with the most consistent and effective anti-viral activity.

Because the epitopes targeted by immunodominant EBV-specific T-cells are most often presented by prevalent HLA alleles, banks of as few as 30-40 3^rd^ party donor EBVCTL lines have provided EBVCTL restricted by one or more HLA alleles shared by a transplant patient for over 95% of patients in ethnogeographically diverse urban populations (11, 44).

The subtypes of CD3+ T-cells and the doses of EBVCTLs administered have not distinguished responders from non-responders in clinical trials thus far reported. Most of the 3^rd^ party EBVCTLs administered have predominantly contained CD8+ T-cells; however, predominantly CD4+ T-cell lines have been similarly effective (11, 44). The number of virus-specific T-cells within the fixed doses of EBVCTLs administered, which have ranged from 2-6 x 10^7^/m^2^ (11, 27, 44–47) have also not distinguished responder from non-responder cohorts. Furthermore, high response rates have been observed even in trials of IFNγ-secreting EBVCTLs separated directly from leukaphereses in which mean doses of only 1.5 x10^5^/m^2^ are administered (38).

## Selection of 3^rd^ party EBV-specific T-cells for treatment of EBVLPD post transplant

Historically, 3^rd^ party EBVCTLs have been selected by choosing EBVCTLs that share the most class I HLA alleles with the patient (28, 38, 48). In SOT patients, this approach has usually worked for two reasons: 1) The EBV+ lymphoma is of patient origin in over 90% of cases (41) and 2) as noted previously, the most immunogenic epitopes of the EBV latency proteins are usually presented by a relatively limited number of prevalent HLA alleles, permitting selection of a partially HLA-matched 3^rd^ party EBVCTL with a high likelihood of activity from a limited bank of seropositive donors most of whom will inherit one or more of the prevalent alleles (49). The same reasoning holds for recipients of HLA-matched related or unrelated HCTs, even though the EBV+ lymphomas developing in HCT recipients are of HCT donor origin in over 90% of cases (10). However, as the therapeutic potential of 3^rd^ party EBV-specific T-cells has become recognized and the number and HLA diversity of patients referred for adoptive cell therapy has increased the need for more precise selection of T-cells based on their HLA restriction and their epitope specificity has become evident. This is especially the case for recipients of HLA haplotype disparate T-cell depleted HCTs or HLA partially matched cord blood grafts administered after conditioning that has included ATG who are at particularly high risk of developing an EBVLPD (13). Again, in such cases, the EBVLPD is usually donor in type. This can pose major challenges to selection of an 3^rd^ party EBVCTL that will target the EBVLPD unless it is known that the EBV-specific T-cells are specific for epitopes on the EBVLPD presented by HLA alleles shared by the transplant donor.

Selection of 3^rd^ party EBVCTLs on the basis of their previously identified HLA restrictions and viral specificities circumvents this problem, permitting the choice of an EBVCTL line restricted by one or more HLA alleles of the transplant donor, host, or optimally both, for immediate treatment of EBV lymphomas of unknown origin. Selection of 3^rd^ party EBVCTLs restricted by alleles shared by both donor and host thereby addresses the two potential origins of the EBV+ lymphoma. In addition, inclusion of EBVCTLs restricted by one or more host HLA alleles may also impose control of EBV in non-lymphoid tissues of the host, such as the salivary glands which may also harbor latent virus that can be reactivated. Pre-identification of the HLA restrictions of each virus-specific T-cells in a bank also permits selection of an EBVCTL restricted by an alternate HLA allele expressed by the EBV+ lymphoma if the initial line fails to induce a response (11, 50).

## Results of trials of HCT donor and 3^rd^ party donor EBV-specific T-cells for post transplant EBVPTLD

Although the number of reported phase I and II trials of EBV-specific T-cells generated *in vitro* from HCT donors or healthy 3^rd^ party donors is still limited, the clinical data accrued provide sufficient evidence to permit initial comparative estimates of their safety and therapeutic activity. The results of trials employing HCT donor-derived EBVCTL are summarized in **Table 1** and those for 3^rd^ party donor-derived EBVCTLs in **Table 2**. Results reported for the use of 3^rd^ party donor-derived EBVCTLs for monoclonal EBV+ lymphomas developing in solid organ transplant (SOT) patients are presented in **Table 3**.

**Table 1:** Results of treatment for biopsy proven rituxan refractory EBVLPD emerging after allogeneic HCT, using HCT donor-derived EBV-specific CTLs.

Center	Method of Sensitization Or Selection	Indication for CTLs	Transplant	Prior Therapy Failed	N	Dose of VSTs	CR + PR %	SD POD	GVH II-IV	Cytokine Storm
**Baylor University, USA** **Heslop et al** (42)	EBVBLCL Sensitized EBVCTLs	PTLD	HCT	None	13	1.5 x 10^7^/m^2^ 3 x 3	11	2		0
**Baylor University, USA** **Leen, AM **(24)	EBVBLCL transduced to also express ADV and CMV antigens-sensitized	PTLD	HCT	None	1	2 x 10^7^/m^2^	1 (100%)	0	0	0
**Pavia, Italy** **Comoli, P **(18)	EBVBLCL Sensitized EBVCTLs	PTLD	TCD HCT	Rituxan	1 Viremia 5 PTLD	0.5 x 10^6^/kg	5 (100%)	0	0	0
**MSKCC, USA** **Doubrovina et al **(10)	EBVBLCL Sensitized EBVCTLs	PTLD	TCD HCT	Rituxan	14	1 x 10^6^/kg x 3	10 (68%)	4	0	0
**Baylor University, USA** **Papadooulou et al** (27)	Multiviral Peptide pool-sensitized VSTs	1 PTLD 2 EBV React	HCT	None	1 2	0.5-2 x 10^7^/m^2^	1(100%) 2(100%)	0 0	0	0
**Baylor University, USA** **Naik, S et al** (37)	Multiviral Peptide Pool Sensitized VSTs	EBV Viremia	HCT	Rituxan 1	6	1-6 x 10^7^/m^2^	6(100%)	0 0	0	0
**Guangzhou, China** **Jiang, Z et al** (49, 51)	Engrafted DCs^+^ EBVBLCL lysate-sensitized EBVCTLs from pts engrafted	PTLD	HCT	RCHOP	8	1 x 10^6^/m^2^	7(88%)	1	4/15	0
**Munich, Germany** **Moosmann, A et al** (30)	IFNγ^+^ peptide react T-cells-imunomagnetic isolation	PTLD	Unmodified HCT	Rituxan	6	0.4-9.7 x 10^:^/m^2^/kg	3(50%)	3	0	0
**Tubingen, Germany** **Icheva, Y et al** (29)	EBNA-1 peptides; IFNγ^+^ Captured T-cells Immunomagnetic isolation	PTLD	Unmodified HCT	Rituxan	8	5 x 10^2^/m^2^ 25 x 10^3^/m^2^	7(88%)	1	1	0
**Baylor University, USA** **Gerdemann, U et al** (25)	EBV, CMV, ADV Plasmid transfected DCs	PTLD3 Viremia	HCT	3 Antiviral Rituxan 9	4	5-20 x 10^6^/m^2^	3(75%)	0	0	0
**Hannover, Germany** **Bonifacius, et al** (31)	EBV Peptide APC IFNγ^+^ T-cell immune magnetic isolation	PTLD	HCT	Chemo 2 Rituxan 9	10	0.5-22 x 10^4^/kg	9(90%)	0 1	2	0
**Budapest, Hungary** **Kallay, K et al** (32)	EBV, CMV, Adv peptides + IFNγ T-cell immunomagnetic isolation	PTLD	HCT	Rituxan 2	2	28-150 x 10^3^/kg	2 (100%)	0 0	0	0

VSTs, Virus-specific T-cells; EBVCTLs, EBV-specific T-cells; EBVBLCL, EBV transformed autologous B –cells.

**Table 2:** Summary of reported HCT experience with adoptive therapy with 3^rd^ party donor derived EBV-CTLs.

Center	Method of Sensitization/ Selection	Indication for CTLs	Prior Therapy Failed	N	HLA Match	Dose of VSTs	CR PR	CR + PR %	SD POD	NE	GVH/Rej	Cytokine Storm
**Edinburgh, Scotland** **Haque et al** (22, 38)	EBVBLCL Sensitized EBV-CTLs	EBV Polymorph Lymphoma HCT	RIS	2	2 – 5/6	2 x 10^6^/kg x4	2 0	100	0 0		0	0
**Alabama, USA** **Sun et al** (44)	EBVBLCL Sensitized EBV-CTLs	Brain	Rituximab	1	6/6	5 x 10^6^/kg	1 0	100	0 0		0	0
**Karolinska, Sweden** **Uhlin et al** (45)	EBV Pentamer Sorted T-Cells	EBV Lymphoma HCT	None	1	5/10	1.8 x 10^4^/kg	1 0	100	0 0		0	0
**MSKCC, USA** **Barker et al** (46)	EBVBLCL Sensitized EBV-CTLs	EBV Lymphoma HCT	Rituximab +/-C	5	>2/10	1 x 10^6^/kg x 5-6	4 0	80	0 1		0	0
**Baylor University, USA** **Leen et al** (34)	Transduced Multivirus/incl EBV-CTL T-cell line	8 EBV PTLD HCT 1 EBV Viremia HCT	Rituximab Rituximab	8 1	≥ 1 1 HLA	2 x 10^7^/m^2^	3 3 0 0	66	0 2 0 1		0	0
**Sydney, Australia** **Withers, B** (28)	Multiviral Peptide pool Sensitized VSTs	NK/T HCT Lymphoma	None	1	1/10	2 x 10^7^/m^2^	0	0	0 1		0	0
**Inserm, E.U.** **Gallot et al** (21)	EBVBLCL Sensitized EBV- CTLs	EBV Lymphoma HCT	Rituximab +/- C	6	≥ 2	5 x 10^6^/kg x 1-3	2 1	50	0 2	1	0	0
**Multi-Center** **Naik et al** (37)	EBVBLCL or DCs transduced to express viral antigens	EBVPTLD HCT Immuno-deficiency	None or Rituximab	5	≥ 3	1-6 x 10^7^/m^2^	1 1	40	0 3		0	1
**Aberdeen, Scotland** **Vickers et al** (47)	EBVBLCL Stimulated EBV-CTLs	EBVPTLD HCT	N/A	6	≥ 3	1-2 x 10^6^/kg	4 0	66	0 2			0
**Baylor University, USA** **Tzannou et al** (35)	EBV Peptide stimulated pool T cell line	EBV PTLD HCT EBV Viremia HCT	None None	1 2	3/8 5/8, 5/8	2 x 10^7^/m^2^	1 2	100 100	0 0		0	0
**MSKCC, USA** **Prockop et al** (11)	EBVBLCL Sensitized EBV-CTLs	EBV Lymphoma HCT	Rituximab	33	≥ 2/10	1-2 x 10^6^/kg x 3	18 3	66	1 9		0	0
**Cincinnati** **Galleta et al** (48)	Multivirus Peptide pool sensitized VSTs	EBV PTLD HCT	NE	9		5 x 10^7^/m^2^						0
**Sydney, Australia** **Jiang, E et al** (49, 51)	EBV peptide pool T cell line	First React of EBV HCT	None	3	2-8/10 1-2/10	2 x 10^7^/m^2^	3 0	100			NE	0
**Hannover, Germany** **Bonifacius A** (31)	EBV PEP Mix pool IFNγ Capture	EBVPTLD HCT	Rituximab	10		5 x 10^3^ -2.2 x 10^5^/kg		60	1 3		1	0
**Ped BMT Consort** **Keller, M** (50)	ADV, CMV EBV Peptide pool T-Cell line	EBVPTLD/LYMPH HCT	Rituximab	4	2-5/10	1.2 x 10^7^/m^2^	11 11	50	1 1		0	0
**Multicenter** **Mahadeo, KM** (41)	EBVBLCL sensitized Tabeleleucel	EBV Lymphoma HCT	CT Rituximab	1 14	≥ 2/10	2 x 10^6^/kg x 3	61 32	50	6 1		0	0
**Multicenter, USA** **Pfeiffer, T et al** (36)	Multivirus peptide pool VSTs	EBV PTLD HCT	Nonspecified	2	NS	2 x 10^7^/m^2^	0 2		0 0		0	0

CT, Chemotherapy; MSKCC, Memorial Sloan Kettering Cancer Center; RIS, reduction in immune suppression; RT, radiation therapy; NS, non-significant; NA, not applicable; NE, Not evaluable.

**Table 3:** Summary of reported SOT experience with adoptive therapy with 3^rd^ party donor derived EBV-CTLs.

Left	Method of Selection	Indication for CTLs	Prior Therapy Failed	N	Dose of VSTs	HLA Match	CR PR	CR + PR %	SD POD	NE	GVH/Rej II-IV	Cytokine Storm
**Edinburgh, Scotland** **Haque et al** (22, 38)	EBVBLCL Sensitized EBV-CTL	EBV Polymorph Lymphoma EBV PTLDSOT	Rituximab	31	2 x 10^6^/kg	2 – 5/6	10 9	61	0 12	0	0	NE
**Alabama, USA** **Sun et al** (44)	EBVBLCL Sensitized EBV-CTL	EBVPTLD SOT Brain	RT Rituximab	1 1	5 x 10^6^/kg	4/6 6/6	1 0 1 0	100	0 0 0 0	0	0	0
**Inserm, E.U.** **Gallot et al** (21)	EBVBLCL Sensitized EBV- CTL	EBVPTLDSOT	C +/- Rituximab	3	5 x 10^6^/kg x 1-3	≥ 2	1 0	33	0 2	0	0	0
**Aberdeen, Scotland** **Vickers et al** (47)	EBVBLCL Stimulated EBV-CTL	EBVPTLDSOT	N/A	4	1.25 x 10^6^/kg	≥ 3	4 0	100	0 0	0		
**Hannover, Germany** **Schultze-Florey et al** (52)	Peptide stimulated INFγ capture	EBV PTLD in remission SOT	CT + Rituximab	1	2.5 x 10^4^/kg x 5	5/10				1		
**Birmingham, UK** **Chiou et al** (53)	EBVBLCL Stimulated EBV-CTL	EBV PTLD SOT	Rituximab	10	1.2 x 10^6^/kg		8 0	80	0 2	0		
**MSKCC, USA** **Prockop et al** (11)	EBVBLCL Stimulated EBV-CTL	EBV PTLD SOT	Rituxan + CT 11/13	13	1.2 x 10^6^/kg	1-6/8	2 5	54	1 5	0	0	0
**Brisbane, Australia** **Gandhi et al** (54)	EBVBLCL Stimulated EBV-CTL	EBV PTLD SOT	Rituxan 3 (+ CT 2)	3	2 x 10^6^/kg	3-4/6	2 0	66	0 1	0	0	0
**Multicenter, USA** **Mahadeo** (41)	EBVBLCL Stimulated EBV-CTL	EBV PTLD SOT	Rituximab or Rituxan + Chemo	29	2 x 10^6^/kg	>2/8	6 9	52	2 7	5		

CT, Chemotherapy; MSKCC, Memorial Sloan Kettering Cancer Center; NA, not applicable; NE, not evaluated.

In comparing the clinical effects and antiviral activity of 3^rd^ party *vs*. HCT donor-derived EBVCTLs in the treatment of EBV+ lymphomas post HCT and also evaluating the activity of 3^rd^ party donor EBVCTLs in SOT recipients, it is important to recognize that the relationship between effector T-cell and tumor target is different. In HCT recipients, the EBV lymphomas that have emerged post transplant have usually been found to be of HCT donor origin. even in those invariably seropositive patients who develop EBVLPD after cord blood grafts or transplants from seronegative donors wherein the transforming EBV strain likely derives from the T-cell ablated patient. In our own series of patients treated with HCT donor or 3^rd^ party donor EBVCTLs, the EBV+ lymphomas were of donor origin in 40/44 cases (91%) (10, 11). Thus, assuming the recipient is engrafted, HCT donor derived EBVCTLs are usually autologous to the tumor cells and should not be rejected. Indeed, genetically marked donor type EBVCTLs have been detected 9 years post infusion (52).

An autologous relationship between SOT patient-derived T-cells and target also applies to the limited number of SOT recipients who have been treated with autologous EBV-specific T-cells generated from their own blood (53–55), since the EBV lymphomas in SOT recipients are of host type in over 90% of cases (41, 56).

In contrast, 3^rd^ party donor-derived EBVCTLs are invariably allogeneic to the HCT donor and host, rendering them susceptible to rejection by functional T-cells in the graft or residual host T-cells surviving pre-transplant conditioning. Likely as a result, these 3^rd^ party EBVCTLs are rarely detected beyond 8-12 weeks post transfer.

In this light, an important finding emerging from the trials summarized in **Tables 1**, **2** is that the initial response rates (CR +PR) achieved with HCT donor-derived and 3^rd^ party donor-derived EBVCTLs are strikingly similar. This is shown in **Tables 1**, **2** for trials at several centers that treated EBV+ lymphomas emerging post HCT with EBV-specific T-cells generated from HCT (10, 24, 31, 36, 37, 39) or 3^rd^ party donors (11, 27, 28, 31, 35, 44–46, 48, 50, 57–63) by the *in vitro* culture methods previously described. Patients treated with EBVCTLs isolated directly by immunomagnetic separation of IFNγ+ T-cells after brief sensitization with pools of immunogenic EBV peptides from latency 1, 2 and 3 proteins (36–39) have also achieved similar response rates even though the doses of EBVCTLs administered were 1-2 log_10_ lower. In our own series in which both HCT donor and 3^rd^ party EBV-specific T-cells were uniformly generated in response to autologous EBVBLCLs, 1 cycle of 3 weekly infusions of HCT donor-derived EBVCTL induced CRs or durable PRs in 68% of patients with Rituximab refractory EBV+ lymphomas. By comparison, of 33 patients with biopsy proven, Rituximab refractory EBV+ lymphomas treated with 3^rd^ party EBVCTLs restricted by HLA alleles shared by the patient, 22 (68%) also achieved CR or durable PR (11). However, maximum responses usually required 2 cycles of EBVCTLs. In another study (38), which included patients treated with either HCT or 3^rd^ party donor IFNγ+ EBV-specific T-cells, the response rate was somewhat higher for recipients of HCT donor-derived T-cells (9/10 *vs*. 6/10). However, clearance of viremia was observed less frequently in recipients of HCT donor-derived EBVCTLs (6/10 *vs*. 8/11). Importantly, initial expansions of EBV-specific T-cells in responding HCT recipients were also comparable in recipients of HCT or 3^rd^ party donor EBVCTLs.

Recipients of SOT who develop EBV+ lymphomas may differ from HCT patients with EBV+ lymphomas at baseline in degree of T-cell deficiency. HCT recipients are usually immunoablated with significantly lower numbers of circulating CD4+ and CD8+ T-cells even when compared to SOT recipients who have failed reduction of immunosuppression as well as CHOP chemotherapy (11). However, SOT patients are often maintained on tacrolimus or cyclosporine following adoptive therapy while HCT recipients are usually not receiving any immunosuppression. Despite these differences, as shown in **Table 3**, response rates for SOT patients receiving 3^rd^ party EBVCTLs for EBV+ lymphomas are also similar to those for HCT patients. However, the range of response rates is broader (11, 27, 28, 48, 51, 57, 60, 64, 65). This variability, in part, likely reflects differences in the degree of immunodeficiency induced in the patients by prior treatment. In certain cohorts, SOT patients have been treated with 3^rd^ party EBVCTLs after failing reduction in the doses of immunosuppressive drugs (RIS) used to prevent rejection of the SOT +/-Rituximab. Response rates in these cohorts have generally been higher than in cohorts treated with EBVCTLs only after the EBV+ lymphoma failed to respond to both Rituximab and an intensive chemotherapy regimen such as CHOP (11, 27, 51). In patients treated by RIS alone or RIS+ Rituximab, 3^rd^ party EBVCTL might be rejected more rapidly; however, recruitment of endogenous T-cells would be expected to be greater resulting in a higher likelihood of clearing the EBV+ lymphomas. In contrast, patients failing treatment with CHOP +/- Rituximab have tended to have more advanced disease by the time they received EBVCTL. Furthermore, they would be expected to be more immunocompromised and reliant on the antiviral activity of the 3^rd^ party EBVCTLs alone to control the EBV+ lymphoma. In our series, 11 of 13 SOT recipients had already failed Rituximab and CHOP. Nevertheless, treatment with 3^rd^ party EBVCTLs proved sufficient to induce a CR or PR in 50% of cases (11).

## Characteristics of transplant donor-derived and 3^rd^ party-derived EBV-specific T-cells that contribute to clearance of EBV+ lymphomas emerging post transplant

Studies of the mechanisms whereby EBV-specific polyclonal T-cells induce regressions of autologous *vs* allogeneic EBV+ lymphomas are limited. However, existing evidence suggests they are initially similar. In the initial clinical trial, Rooney et al (23) intravenously infused EBV-specific HCT donor T-cells transduced to express a neomycin resistance gene as a reporter, and showed, in two patients, that these EBVCTLs were subsequently detected in each patient’s responding EBV+ lymphoma (23, 50). In contrast, no marked EBVCTLs were detected in another patient’s pre-treatment sites of chronic GVHD (23). In a subsequent report of this clinical trial, Heslop et al. (66) further demonstrated that HCT donor-derived EBVCTL expressing the neomycin reporter gene increased in the blood following infusion into HCT patients and persisted for 18 months post infusion.

In immunodeficient mice bearing human EBVBLCL xenografts, we early observed that HLA A*0201 restricted EBV-specific T-cells administered intravenously preferentially homed to autologous EBV+ tumors and induced their clearance (67). Subsequently, we showed that in mice simultaneously bearing an autologous and 3 allogeneic subcutaneous tumor xenografts, HLA-restricted EBVCTLs selectively homed to and induced complete regressions of both the autologous and a 3^rd^ party EBVBLCL xenograft bearing the EBVCTLs’ restricting HLA-A0201 HLA allele, but did not migrate to or alter the growth of either an allogeneic HLA-A0201 negative EBVBLCL or an EBV^-^ HLA A0201+ allogeneic B-lineage ALL (68). Furthermore, none of the mice showed evidence of xenogeneic GVHD (68).

Taken together, these early studies demonstrated the potential of *in vitro* generated allogeneic 3^rd^ party EBVCTL to expand *in vivo*, selectively target autologous and allogeneic EBV transformed cells expressing their restricting HLA allele and thereby induce regressions of the targeted EBV+ tumors without inducing or requiring non-specific, xenospecific or allo-specific activity to achieve an anti-tumor effect. Such selective targeting of both HCT and 3^rd^ party donor-derived HLA-restricted EBV-specific T-cells is central to their mechanism of action, their safety, their broad and successful clinical application as well as a significant proportion of their failures.

## Clinical responses to infusions of HCT or 3^rd^ party donor derived EBVCTLs

### Safety

As shown in **Tables 1**–**3**, the infusions of transplant donor and third-party donor derived EBVCTLs have been consistently well tolerated, with minimal if any incidents of severe acute toxicities clearly associated with the administration of the T-cells. The incidence of grade 2-4 acute or chronic GVHD has also been negligible. Furthermore, no instances of organ graft rejection have been recorded. In addition, the cytokine release syndrome and the immune cell associated neurotoxicity syndrome (ICANS) that have been a significant and potentially lethal complication of adoptive therapy with autologous T-cells transduced to express a CD19-specific CAR after non-specific activation T-cells (69, 70), have rarely been observed following infusion of virus-specific T-cells (VSTs), be they specific for EBV alone (71) or for EBV and other viruses such as CMV, adenovirus or BK virus (71). The basis for this difference in risk is unclear. However, our center has found that EBV-specific T-cells transduced to express the same CD19 specific CAR construct used to transduce unselected T-cells non-specifically activated by αCD3 and αCD28 rarely induce clinically detectable CRS (71) suggesting that the viral specificity of the EBV-specific T-cells pre-selected for CAR modification may limit their capacity to induce systemic off target reactions.

### Initial clinical responses

The first clinical evidence of a therapeutic response to HCT donor-derived EBVCTLs can be seen clinically as early as 5-7 days post initial infusion with resolution of fever or a noticeable reduction of the size of a palpable lymph node. In recipients of 3^rd^ party EBVCTL, this usually occurs somewhat later (8-15 days) (11). Although some responders may exhibit a brief spike in the levels of EBVDNA, these levels usually decrease to below pre-infusion levels by 7-10 days in responding recipients of either HCT donor or 3^rd^ party EBVCTLs (10, 27, 31, 32, 36–39, 48, 57, 72). Responders also consistently exhibit increases in the level of EBV-specific T-cells in the blood (10, 11, 31, 32, 36–38, 44, 48, 57, 72, 73). Radiologic improvements may be seen by 3 weeks in responding recipients of HCT donor EBVCTLs and usually by 5 weeks in all responders. Among recipients of 3^rd^ party EBVCTL, we identified a proportion of patients with stable disease and stable or less than 2 x log10 reductions in EBV DNA levels, when assessed 5 weeks after initial infusion. These patients also had small but detectable increases in EBVCTL levels (11, 32, 38). Such patients can often achieve CR or durable PR with additional cycles of the same EBVCTLs (11). In contrast, patients with progressive disease by 3-5 weeks post initial infusions (11, 31, 36, 37, 72) warrant a switch to treatment with EBVCTLs restricted by a different HLA allele or other approaches.

### Persistence of responses

A surprising feature of the responses observed in HCT recipients treated for biopsy proven EBV lymphomas with 3^rd^ party EBVCTLs has been the persistence of the responses and the extremely low incidences of recurrence observed (10, 41, 66). In HCT patients treated with transplant donor-derived EBVCTLs, such persistence has been regularly observed, and ascribed to the fact that patients who already received an HCT from the donor and are engrafted would not be expected to reject EBVCTLs from the same donor. Indeed, Heslop et al. (52) has reported that EBVCTLs transfected to express a neomycin resistance gene marker could be subsequently detected in such patients as late as 9 years post infusion (52). In contrast, 3^rd^ party EBVCTLs are allogeneic to the patient and HCT donor and are expected to be rejected. In fact, despite the immunodeficient state of HCT recipients who have developed EBVLPD in the first 3 months post transplant, persistence of 3^rd^ party EBVCTLs has rarely extended beyond 12 weeks post infusion (11, 44). Furthermore, even in patients with partial responses to 3^rd^ party EBVCTLs, lesions have continued to slowly resolve and levels of EBV DNA have remained low without additional therapy, indicating sustained control of the EBV proliferative disease (11, 44). The persistence of 3^rd^ party EBVCTLs in SOT recipients is similar (11, 48). However, in 2 reported SOT patients on chronic immunosuppressive drugs, 3^rd^ party EBVCTLs have been detected as late as 17 months and 22 months post infusion (11, 51).

The mechanisms contributing to the persistence of responses to 3^rd^ party EBVCTLs are still poorly understood. However, growing evidence suggests that while initial clinical and pathological responses reflect the activity of the infused 3^rd^ party EBVCTLs, these responses are likely principally sustained by endogenous EBVCTLs generated from the HCT donor or residual T-cells of the host. Sequential analyses can demonstrate persistence of circulating EBV-specific T-cells in the blood of recipients of EBVCTLs derived from 3^rd^ party or HCT donors (11, 38, 44, 62). To identify the origin of these circulating EBVCTLs several approaches have been employed. The first, based on sequencing of the EBV-specific TCRs in the infused EBVCTLs can identify TCRs present in the VST product prior to infusion and track them *in vivo* after infusion either by deep sequencing or by expansion followed by deep sequencing (38, 44). The second, based on the use of unique STRs (11, 74), has the advantage of being able to distinguish 3^rd^ party donor-derived populations of EBV specific T cells emerging after infusion from those originating from the patient (either from the recipient or original stem cell donor). Using digital droplet PCR methods of detection of unique STRs, intermittent very low level detection has been identified (62) for a relatively short period of time after infusion despite the durability of responses. In our studies, 3rd party EBVCTLs have been regularly detected in the first 4-5 weeks post infusion (11) similar to what others (38, 44) have reported based on the TCR sequencing approach. However, thereafter, the circulating EBVCTLs have usually been predominantly or exclusively of HCT donor (or in some instances recipient) type suggesting that infusion of 3^rd^ party EBVCTLs may foster expansion of recipient populations. There is also evidence for the interaction of infused populations with the recipient in the autologous setting. Using deep sequencing TCR methods several groups have shown emergence of populations with unique TCRs including T cells recognizing tumor antigens after infusion of autologous viral specific T cells targeting both CMV (75, 76) and EBV (77) suggesting that this process may not depend on allorecognition.

## Clinical factors contributing to the success or failure of 3^rd^ party EBVCTLs in the treatment of EBV+ lymphomas post transplant

The clinical factors affecting treatment outcome can be principally divided into 3 categories: 1) Clinical features of the patient; 2) characteristics of the EBV lymphoma at time of treatment and 3) the functional properties and specificities of the 3^rd^ party EBVCTLs targeting the patient’s EBV+ lymphoma.

Although the data are still limited, certain patient characteristics have been associated with a lower probability of a CR or PR following treatment with 3^rd^ party EBVCTLs. It is tempting to assume the patient’s baseline level of functional T-cells plays a significant role since recipients of T-cell depleted hematopoietic cell grafts and cord blood grafts are at significantly higher risk of developing an EBVLPD (13, 78). However, in our trials, we have observed that, in contrast to HCT recipients treated for drug refractory CMV infections in whom higher levels of T-cells prior to treatment with 3^rd^ party CMVCTLs have been associated with a significantly higher likelihood of response (11, 79), among HCT recipients treated for an EBV+ lymphoma, this advantage was not detected (11). This may reflect the fact that in our series, levels of T-cells/µL detected in both responders and non-responders in the cohort treated for EBV+ lymphoma were significantly higher than those in patients who failed to respond to CMVCTLs and were similar to those detected at baseline in HCT recipients with drug refractory CMV infections who responded to CMV-specific 3^rd^ party CTLs.

Because most trials of adoptive therapy have excluded patients with grade II-IV acute GVHD, the effects of GVHD or its treatment on the outcome of adoptive therapy are unclear. However, pre-transplant conditioning that included ATG has been cited not only as a risk factor for development of EBVPTLD (13) but also a risk factor for a lower response rate to 3^rd^ party multivirus specific T-cells (63). Ongoing treatment with systemic corticosteroids has been variably associated with lower responses to virus-specific T-cells in patients treated for CMV and adenovirus infections, but too few patients on steroid have been treated for EBVPTLD to clearly assess significance. The impact of different doses of steroids on the proliferation and antiviral activity of virus-specific T-cells needs close evaluation since they are a mainstay of treatments for GVHD or organ rejection. Most trials of adoptive therapy for viral diseases have already excluded patients receiving ≥0.5 mg/kg prednisone or its equivalent. Thus, the lower responses reported suggest that even lower, as yet undefined, doses may influence responses.

In contrast to steroids, our own and other trials have found that ongoing treatment with cyclosporine, tacrolimus or mycophenolate has not affected response rate, nor have these immunosuppressive drugs prevented expansion of EBVCTL populations in these patients (11, 39, 62). *In vitro*, mycophenolate has been found to primarily inhibit the growth and differentiation of naïve antigen-specific T-cells, and has limited activity against pre-existing memory T-cells (80, 81). Tacrolimus can inhibit generation of immunostimulatory cytokines (i.e. IL-2, IFN and TNF). They will also delay but do not prevent proliferation of virus-specific T-cells or inhibit their cytotoxic functions (80, 81). Thus, the *in vivo* findings that they do not prevent the expansion of adoptively transferred EBVCTL or their antiviral activity *in vivo* is consistent with their limited activity against the memory T-cells that constitute the bulk of those T-cell populations.

Several clinical characteristics of the EBV+ lymphoma may influence its response to 3^rd^ party EBVCTLs. While no differences in response have been correlated with the pathologic type of EBV+ monoclonal PTLD treated (11) the extent and severity of the EBV infections is a principal determinant. Thus, patients treated preemptively upon detection of EBV viremia have rarely failed to clear viremia or developed EBVLPD following infusions of 3^rd^ party EBVCTLs with reported response rates of 83-100% (16, 17, 62). Similarly, those with biopsy proven EBV+ lymphoma post HCT, with only 1-2 sites of disease confined to nodal areas fare significantly better than patients with EBV+ lymphomas involving ≥ 3 sites (11, 82). Patients with extranodal sites of EBV+ lymphoma also have significantly lower rates of CR +PR than those without (11, 82). However, one extranodal site where EBV+ lymphomas have exhibited striking sensitivity is the CNS. In the initial trial of Haque et al. (48), three of four SOT recipients with EBV+ lymphomas in the CNS achieved a CR or PR. In our trial, 9/11 patients (6 HCT, 5 SOT) with EBV+ lymphoma lesions in the CNS have achieved a CR or sustained PR (11) following infusions of 3^rd^ party EBVCTLs. Other centers have also recorded good response rates for CNS lesions (51, 57, 83, 84).

Prior treatment of the EBV+ lymphoma can also affect response rates, since those that have failed to respond to both Rituximab and chemotherapy are less likely to respond to 3^rd^ party EBVCTLs than those that have failed Rituximab alone (11). Such chemotherapy may reduce the potential of 3^rd^ party EBVCTLs to recruit endogenous T-cells to contribute to anti-viral responses.

## Characteristics of 3^rd^ party donor-derived EBV-specific T-cells and the transplant recipient’s EBV+ lymphoma that affect their interaction and treatment outcome

To be effective as an immunotherapeutic, 3^rd^ party donor-derived EBVCTLs must be able to recognize and engage the EBV+ lymphoma, proliferate sufficiently and be able to kill the EBV+ lymphoma cells and reestablish control over residual latently infected lymphoid populations.

EBV has developed a strikingly varied and complex array of mechanisms whereby it can establish latency and evade the host immune system. These have recently been extensively reviewed (85–87). However, certain features of EBV+ lymphomas emerging post transplant that distinctively prevent their recognition and effective engagement by allogeneic 3^rd^ party EBV-specific T-cells have also been observed. These warrant particular attention since they can contribute to treatment failures, and in a proportion of patients, can be clinically overcome. These are listed in **Table 4** and can be briefly described.

**Table 4:** Distinctive mechanisms whereby EBV+ lymphomas emerging following HCT or SOT evade 3^rd^ party EBV-specific T-cells.

HLA-restriction EBV-specific T-cells are specific for an epitope presented by an HLA allele not shared by the EBV+ lymphoma:
• The EBV+ lymphoma is derived from the HCT recipient rather than the donor/or the SOT donor rather than the recipient.
• The HLA allele or the EBV+ lymphoma by which the EBV-specific T-cells are restricted is downregulated by the virus.
• The HLA haplotype encoding the HLA allele by which the EBV-specific T-cells are restricted is deleted.
EBV epitopes targeted by the EBV-specific T-cells are not presented:
• Epitopes that are usually dominant for a specific HLA allele are not the epitopes that induce the EBV-specific T-cells in the T-cells used for treatment
• The EBV epitope targeted is mutated.
• The EBV epitope targeted by the EBV-specific T-cells is not presented adequately by the EBV strain inducing the EBV lymphoma due to limited expression.
• The EBV epitope targeted by the EBV-specific T-cells is not presented due to impaired processing and/or presentation induced by EBV evasins.

The origin of EBV+ lymphomas emerging post transplant is often not identified at time of referral. Although 90% of EBV+ lymphomas in HCT patients are of donor type (10, 11) and 90% in SOT recipients are host type (41, 56), this is not always the case. When the origin is unknown, selection of EBVCTLs restricted by an HLA allele shared by both transplant donor and host is indicated. We have observed such case in a child who developed an EBVLPD following an HCT from his HLA-haplotype disparate parent. EBVCTLs generated from this parent were restricted by an HLA allele not shared by the host type EBV lymphoma. The tumor progressed and was controlled only after treatment with 3^rd^ party EBV-specific T-cells restricted by an HLA allele shared by the patient (10).

Another, poorly understood mechanism for preventing T-cell recognition of EBV antigens presented by HLA alleles is the emergence of EBV lymphoma clones exhibiting loss of HLA heterozygosity resulting from deletion of the chromosomal 6p segment and development of uniparental disomy. This has been observed frequently in EBV+ nasopharyngeal carcinomas (88). In patients receiving HLA haplotype disparate HCTs for AML, this has been observed in up to 30% of the AMLs that have relapsed post transplant. Strikingly, the HLA haplotype lost has been the haplotype unique to the host’s AML (89, 90). Recently, we have observed uniparental disomy in an EBV+ lymphoma that developed in a recipient of a double cord blood transplant (CBT) in which the HLA haplotype deleted was the haplotype of the non-engrafted CBT that was not shared by either the patient or the engrafted CBT (unpublished).

Several EBV-derived proteins and microRNAs have also been shown to downregulate expression of HLA alleles (85–87, 91). This would be expected to inhibit or prevent engagement by HLA restricted virus-specific T-cells. However, clinical instances in which selective downregulation of an HLA allele has played a role in blocking the targeted activity of adoptively transferred 3^rd^ party. EBVCTLs have thus far not been reported.

Characteristics of the specific virus causing the EBV+ lymphoma may also prevent recognition by appropriately HLA restricted EBV-specific T-cells.

Differences between EBV strains causing the EBV+ lymphoma and the B95.8 EBV strain used to produce EBVBLCLs for sensitization and generation of virus-specific T-cells may also limit T-cell effectiveness. For example, in 3 patients who developed HCT donor type EBV+ lymphomas and failed treatment with HCT donor derived, B95.8 strain EBVBCL sensitized T-cells, we found that while the B95.8 EBV sensitized T-cells failed to lyse the spontaneous EBV+ BLCL isolated from the patient’s tumor or blood, T-cells from the same patient sensitized with the spontaneous transformants from the patient lysed both the autologous HCT donor-type B95.8 transformed and the spontaneously transformed HCT donor-type B cells grown from the patient’s tumor (10). These cases suggested that a dominant antigen was presented by the patient’s EBV strain that could stimulate generation of effective virus-specific T-cells, but that antigen was either not expressed or not presented adequately to elicit a T-cell response by B cells of the same donor origin that had been transformed by the EBV B95.8 strain.

Similarly, certain of the latency 1,2 and 3 proteins may have mutations that can alter or delete EBV peptide epitopes targeted by the EBVCTLs, rendering the T-cells ineffective. For example, Gottschalk et al. (92) have reported a patient who failed treatment in whom spontaneously transformed EBV+ B cells had a mutation in EBNA3B resulting in deletion of 2 epitopes presented by HLA A*1101 targeted by the T-cells used for treatment.

Failure of appropriately HLA restricted EBVCTLs to recognize an EBV+ lymphoma may also reflect rapid clearance of the EBVCTLs post infusion, intrinsic failures of the EBV lymphoma cells to express specific proteins, or virus-induced alterations in the processing of immunogenic EBV peptides or their presentation by specific HLA alleles. For example, we have reported a patient with an EBV+ lymphoma of transplant donor origin that normally expressed HLA A*1101 who failed to respond to 3 separate HLA A*1101 restricted EBNA3B-specific 3^rd^ party EBVCTLs but did respond to EBVCTLs restricted by another HLA allele expressed by the lymphoma, HLA B*4403 (11). In this case, malignant EBV+ B cells were grown from the tumor. The latency proteins EBNA-1, 2, 3A, 3B, 3C, LMP-1 and LMP-2 from the EBV in the tumor were sequenced, and peptides known to be presented by HLA A*1101 compared to those for the same peptides from strain B95.8 used for T-cell sensitization. They were identical, including those of the EBNA3B epitopes identified to be targeted by the 3 HLA A*1101 restricted EBVCTLs. This is particularly important since EBNA3B has been shown to be a virus-encoded tumor suppressor and EBV strains lacking EBNA3B or expressing inactivating mutations thereof have been found to promote EBV-induced lymphomagenesis and to suppress tumor infiltration by T-cells (93). Furthermore, both HLA A*1101 and EBNA3B proteins were fully expressed by the EBV+ lymphoma. Despite these findings the HLA A*1101 restricted EBVCTLs failed to lyse the patient’s tumor cells *in vitro* or alter disease progression *in vivo*. In contrast, the HLA B*4403 restricted EBVCTL lysed the tumor cells expanded *in vivo* and induced a durable CR (11). We cannot rule out the possibility that the only dominant epitopes expressed on this patient’s tumor were presented by HLA B*4403 or other alleles unique to the patient. However, with the exception of HLA B*4403, EBVCTLs restricted by the other HLA alleles unique to this tumor were not identified in our bank of over 300 EBVCTL lines (11). In contrast, several lines specific for the targeted peptides presented by HLA A*1101 were. For these reasons, the failures of the HLA-A*1101 restricted EBVCTLs seem more consistent with EBV induced impairment of antigen processing and/or presentation. Such impairments may be induced by one or more of the EBV evasin proteins and miRNAs that, either broadly or in an HLA allele selective manner can inhibit antigen processing and TAP-mediated transfer of the viral peptide epitopes to their presenting HLA allele (94–96).

## Current approaches to address those HCT and SOT patients with a rituximab refractory EBV+ lymphoma who fail to respond to initial treatment with 3^rd^ party EBV-specific T-cells

A major and currently accessible advantage to the use of 3^rd^ party EBV-specific T-cells of defined HLA restrictions is that patients failing to respond to initial treatment with T-cells specific for an EBV epitope presented by one HLA allele can be switched to another 3^rd^ party EBVCTL restricted by an alternate shared HLA allele. This approach can circumvent several of the obstacles to effective T-cell recognition of EBV+ tumor targets, including the impaired antigen processing and presentation induced by certain EBV evasins if, as is the case for CMV (97), the evasins selectively affect presentation by certain class I alleles and not others. In early clinical trials, this “switch therapy” has secured CRs or durable PRs for 40-60% of patients whose EBV+ lymphoma failed to respond to an initial 3^rd^ party EBVCTL line (11, 50).

Epigenetic modifiers such as 5azacytidine, gemcitabine and others can also alter the latency of EBV+ malignant cells, thereby inducing EBV-associated tumors expressing only latency 1 or latency 2 EBV proteins to express the array of latency 3 and lytic phase proteins of EBV (98, 99). As shown in a clinical trial by Perrine et al. (100), induction of a lytic cycle of EBV by one such agent, arginine butyrate, can render EBV+ lymphomas in SOT patients sensitive to the viral TK inhibitor ganciclovir in a proportion of cases. Furthermore, in murine models, even a limited *in vivo* exposure to gemcitabine can induce prolonged expression of latency 3 proteins and presentation of their processed antigens, resulting in susceptibility to 3^rd^ party T-cells specific for these antigens both *in vitro* and *in vivo* (99).

As early shown by Pule et al. (94), EBV-specific T-cells when genetically modified to express a chimeric antigen receptor (CAR) specific for a differentially expressed antigen such as GD-2, can induce regressions of GD-2+ tumors such as neuroblastoma. In murine model systems and early clinical trials, 3^rd^ party EBV T-cells expressing a CAR specific for the B-cell antigen CD19 have also been effective in inducing remissions of EBV negative B cell lymphomas (71, 95). In transplant recipients, 3^rd^ party CD19 CAR+ EBVCTLs could also have significant advantages: First, the CAR T-cells are not HLA restricted. Second, they are depleted of alloreactive T-cells that could cause GVHD or rejections of an organ allograft. Third, treatment with these CD19CAR+ EBVCTLs has not been associated with cytokine release syndromes.

Because of the lower immunogenicity of LMP-1, and LMP-2 compared to EBV latency 3 proteins HCT donor and 3^rd^ party donor EBVCTLs generated in response to EBVBLCL have usually contained a predominance of T-cells specific for peptides from latency 3 EBV proteins. However, the recent demonstrations of the potential of autologous T-cells selectively sensitized with immunogenic peptides of LMP-2 and LMP-1 to induce remissions in heavily pretreated patients with EBV-associated malignancies such as EBV+ nasopharyngeal carcinoma and Hodgkin’s disease (77, 96, 101) and the use of HCT donor-derived LMP-specific CTLs to reduce the incidence of relapse in patients receiving allogeneic HCTs for EBV associated B cell lymphomas and NK/T cell lymphomas (102), suggest 3^rd^ party T-cells specific for LMP-1 and LMP-2 might also be effective in the treatment of PTLD. This approach is currently in clinical trials. Whether such T-cells would exhibit activity comparable to that of currently used 3^rd^ party EBVCTLs remains to be seen.

Another approach which has already been introduced for the treatment of certain EBV-negative malignancies is the use of T-cells transduced to express TCRs specific for a tumor-unique or differentially expressed antigen. Promising examples include clinical trials of autologous T-cells transduced to express a MART-1/HLA A0201-specific TCR for treatment of HLA A0201+ patients with melanoma or a NY-ESO-1/HLA-A0201-specific TCR to treat synovial sarcomas (103, 104). HCT donor-derived T-cells transduced to express a WT-1/HLA A0201-specific TCR have also induced or sustained CRS in a limited proportion of patients treated for relapse of acute myelogenous leukemia post transplant (105).

Development of TCR-modified T-cells for treatment of EBV malignancies has focused on the generation of T-cells transduced to express TCRs specific for the latency II EBV antigens LMP-1 and LMP-2 as well as EBNA-1 since these antigens are selectively expressed by much more prevalent EBV-associated malignancies like EBV+ nasopharyngeal carcinoma as well as EBV+ Hodgkin and non-Hodgkin lymphomas and the often refractory EBV+ NK/T-cell lymphoma (106). Indeed, adoptive transfer of autologous T-cells sensitized with APCs transduced with adenoviral vectors encoding LMP1 and LMP2 has induced responses (CR+PR) in over 50% of patients with chemotherapy refractory EBV+ Hodgkin’s disease and 4/6 patients with EBV+ NHL (77, 101).

Initial analyses of T-cell responses to autologous EBV transformed B-cells and, thereafter, to pools of peptides derived from EBV proteins expressed during latency phases 1-3 led to identification of immunodominant peptide epitopes presented by several prevalent HLA alleles (49, 107). More recently, studies of LMP-1, LMP-2 and EBNA-1 epitope/HLA specific T-cells segregated by their binding to peptide/MHC multimers has facilitated the isolation and expansion of clones with high affinity. Subsequent sequencing of their TCR CDR3 genes has led to identification of variable numbers of TCR clonotypes specific for individual epitopes. Examples include TCRs specifics for epitopes of LMP-1 presented by prevalent HLA alleles such as HLA A*0201 (108, 109) and HLA A*1101 (110, 111) as well as rarer alleles such as HLA B*5701 and HLA C*1502 (112). Using a similar approach, TCRs specific for both latency 3 and lytic EBV antigens have also been sequenced (112–117). In sequential analyses of HCT-donor-derived epitope-specific T-cell clonotypes following transplant (40) Lommoglia Cobo et al. have also demonstrated differential expansions and persistence of certain immunodominant clonotypes detected in the transferred cells and not others. This study (40) further demonstrated diversification of the repertoire of EBV epitope-specific T-cell clonotypes both from those uniquely detected in the adoptively transferred populations and from the host. A similar expansion and a diversification of autologous CMV-specific T-cells from both transferred and endogenous clonotypes has been reported by Smith et al. (43) for SOT patients with drug-refractory CMV infections who responded to adoptive therapy.

Recently, several groups have developed novel retroviral-based constructs for transducing adult or cord blood T-cells to express activatable TCRs specific for immunodominant epitopes of selected EBV proteins and have begun pre-clinical assessments of their activity against EBV+ malignancies (109–112, 117–119). However, while techniques securing efficiently transduced T-cells that highly express a correctly paired, activatable, high affinity TCR that can induce epitope-specific HLA restricted lysis of EBV+ tumor cells *in vitro* are increasingly effective, selection of one or a set of epitope-specific TCRs that, when expressed by autologous or an allogeneic donor’s T-cells will ensure therapeutic efficacy is a major challenge. At issue is whether and to what degree T-cell clonotypes specific for immunodominant epitopes that differentially expand in responding patients *in vivo* can be identified as clonotypes that consistently produce a tumor response. Alternatively, would sets of T-cells bearing different TCRs against one immunodominant epitope or against epitopes presented by more than one HLA allele be more effective. At present, there is some evidence supporting selections of T-cells expressing the rare, so called “public TCRs” (i.e. TCRs that are detected in more than one donor) that regularly exhibit high affinity for their epitope/HLA targets and function well *in vitro* against EBV+ cells expressing the epitope (117, 120). However, as the TCRs of EBV epitope-specific T-cells generated from 3^rd^ party donors that have been used to treat more than one patient are identified by their Vα and Vβ sequences, functionally characterized and followed in patients post adoptive therapy, it should be possible to better select T-cell clonotypes for adoptive therapy that are consistently effective.

In conclusion, banks of 3^rd^ party donor derived EBV-specific T-cells sensitized with autologous EBVBLCLs or other antigen-presenting cells loaded with pools of immunogenic peptides derived from latency 1, 2 and 3 EBV proteins alone or together with other viral peptides can provide immediately accessible T-cells of defined viral specificity and HLA restriction for treatment of EBV+ lymphomas emerging after HCT or SOT. In clinical trials, these 3^rd^ party EBVCTLs have been safe, and associated with minimal incidences of GVHD, graft rejection, or cytokine release syndromes. These 3^rd^ party EBVCTLs have also induced responses (CR + PR) in Rituximab resistant EBV lymphomas at rates that are thus far comparable to those achieved with HCT donor derived EBV CTLs. Furthermore, similar rates of CR + PR and persistence of responses have been achieved in the treatment of Rituximab and chemotherapy refractory EBV+ lymphomas in SOT recipients. The reasons for treatment failures are complex. While clinical features of the patient and the EBV+ lymphoma significantly influence the rates of response, most treatment failures principally reflect the failure of adoptively transferred EBVCTLs to recognize and effectively engage the EBV+ lymphomas either because the T-cells are restricted by an HLA allele not shared by the EBV+ lymphomas or are specific for an antigen or antigens that are not adequately expressed or presented by the tumor cells. Early clinical results provide evidence that switching to 3^rd^ party EBVCTLs specific for an alternative antigen presented by a different shared HLA allele can induce CRs or durable PRs in such cases. Preclinical data and early trials also suggest that enhancement of viral antigen expression on tumor cells targeted by the EBVCTLs or the use of CAR-modified EBV-specific T-cells might also be effective for treatment of EBV+ lymphomas failing initial EBVCTL therapy. Current trials of early preemptive therapy with EBV-specific or multivirus-specific 3^rd^ party T-cells also show promise of significantly further reducing the incidence, morbidity and mortality of EBV+ lymphomas following either HCT or SOT.

## Author contributions

RO: Writing – original draft, Writing – review & editing. SP: Writing – review & editing. JO: Writing – review & editing.
